# Identification of Candidate Reference Genes in Perennial Ryegrass for Quantitative RT-PCR under Various Abiotic Stress Conditions

**DOI:** 10.1371/journal.pone.0093724

**Published:** 2014-04-03

**Authors:** Linkai Huang, Haidong Yan, Xiaomei Jiang, Guohua Yin, Xinquan Zhang, Xiao Qi, Yu Zhang, Yanhong Yan, Xiao Ma, Yan Peng

**Affiliations:** 1 Department of Grassland Science, Animal Science and Technology College, Sichuan Agricultural University, Ya’an, Sichuan, China; 2 College of Agriculture and Life Sciences, The University of Arizona, Tucson, Arizona, United States of America; 3 National Animal Husbandry Service, Ministry of Agriculture, Beijing, Beijing, China; Northwestern University, United States of America

## Abstract

**Background:**

Quantitative real-time reverse**-**transcriptase PCR (qRT-PCR) is an important technique for analyzing differences in gene expression due to its sensitivity, accuracy and specificity. However, the stability of the expression of reference genes is necessary to ensure accurate qRT-PCR assessment of expression in genes of interest. Perennial ryegrass (*Lolium perenne* L.) is important forage and turf grass species in temperate regions, but the expression stability of its reference genes under various stresses has not been well**-**studied.

**Methodology/Principal Findings:**

In this study, 11 candidate reference genes were evaluated for use as controls in qRT-PCR to quantify gene expression in perennial ryegrass under drought, high salinity, heat, waterlogging, and ABA (abscisic acid) treatments. Four approaches – Delta C_T_, geNorm, BestKeeper and Normfinder were used to determine the stability of expression in these reference genes. The results are consistent with the idea that the best reference genes depend on the stress treatment under investigation. Eukaryotic initiation factor 4 alpha (*eIF4A*), Transcription elongation factor 1 (*TEF1*) and Tat binding protein**-**1 (*TBP-1*) were the three most stably expressed genes under drought stress and were also the three best genes for studying salt stress. *eIF4A*, *TBP-1*, and Ubiquitin**-**conjugating enzyme (*E2*) were the most suitable reference genes to study heat stress, while *eIF4A*, *TEF1*, and *E2* were the three best reference genes for studying the effects of ABA. Finally, Ubiquitin (*UBQ*), *TEF1*, and *eIF4A* were the three best reference genes for waterlogging treatments.

**Conclusions/Significance:**

These results will be helpful in choosing the best reference genes for use in studies related to various abiotic stresses in perennial ryegrass. The stability of expression in these reference genes will enable better normalization and quantification of the transcript levels for studies of gene expression in such studies.

## Introduction

Perennial ryegrass (*Lolium perenne* L.) is dominant forage and turf grass specie in temperate regions due to its good grazing tolerance, extraordinarily high digestibility and adequate seed production [1], [2]. Perennial ryegrass is cultivated in the USA, China, Japan, UK, Australia, New Zealand, South Africa and South America [3], [4]. The species may also aid China in mitigating food shortages issue since it may be capable of producing marketable yields on marginal agricultural land. However, marginal lands are usually afflicted by abiotic stresses such as heat, cold, drought, waterlogging and high salinity, all of which may negatively impact yield, quality, and growth of perennial ryegrass. For example, perennial ryegrass is a drought**-**susceptible grass species [5] and its leaf extension and appearance rates are both reduced under drought stress [6]. Under waterlogging treatment, the emergence and seedling growth of cv. S.24 perennial ryegrass is significantly reduced in a glasshouse environment [7], with dry matter yield reduced by up to 25% [8]. Moreover, as a cool season grass, perennial ryegrass has poor heat resistance, making it more likely to die in high**-**temperature, and high**-**humidity regions in summer. The susceptibility of perennial ryegrass to freezing temperature also limits its cultivation [9].

Understanding the genetic responses of different varieties of perennial ryegrass to differential abiotic stresses could lead to better germplasm utilization strategies for successful agronomic production in regions experiencing different climate conditions. For example, the stress tolerance**-**conferring miR398 could potentially be used as a genetic component or a genetic marker for plant stress tolerance [10]–[12]. Numerous studies clarifying genetic mechanisms of abiotic stress responses in plants have been based on gene expression analysis [13]–[15]. In perennial ryegrass, the expression of C**-**repeat (CRT) binding factors (*CBF*) gene can be rapidly induced in response to low temperature [16], while expression of myoinositol 1**-**phosphate synthase (EC 5.5.1.4) and galactinol synthase (EC 2.4.1.123) genes can be decreased by drought stress [17]. Therefore, the exploration of expression patterns of candidate stress tolerance gene is critical to illustrate the mechanisms underlying abiotic stress tolerances in perennial ryegrass.

Quantitative real**-**time reverse**-**transcriptase PCR (qRT-PCR) together with Northern blots, microarrays, and RNA**-**Seq analysis [18] are useful for inferring the transcription levels of genes, and especially for detecting low-quantity mRNAs. In particular, qRT-PCR is valuable for its accurate quantification of target genes across a relatively broad dynamic range [19], [20]. However, numerous factors –RNA stability, quality, or quantity, transcription efficiencies, and PCR reaction conditions – can all affect the reliability of qRT-PCR. In order to ensure that any variation observed in qRT-PCR estimates of transcript levels are due to changes in expression of the target gene (s) rather than overall variations in mRNA among biological samples or replicate, multiple genes should be analyzed simultaneously. To avoid bias, qRT-PCR is also typically referenced to an internal control gene having relatively stable expression for comparison to the target genes [21].

Reference genes maintain stable gene expression based on the need to support cell function or cell structure across various experimental conditions, and can contribute to the identification of subtle differences in expression among genes of interest [22], [23]. Numerous reference genes are commonly used in gene expression studies, such as β**-**actin (*ACT*), glyceraldehyde**-**3**-**phosphate dehydrogenase (*GAPDH*), translation elongation factor (*TEF*), tubulin (*TUB*), ubiquitin conjugating enzyme (*UBC*), 18S ribosomal RNA (*18S rRNA*), and 25S ribosomal RNA (*25S rRNA*) [24]–[26]. Nevertheless, some of these reference genes may differ in expression among plant tissues, species and growth conditions (e.g., abiotic stresses). If this is the case, a study aiming to compare gene across such conditions may have no reference genes with a suitably stable expression profile [27]–[30], making the unbiased assessment of target genes difficult. Therefore, it is crucial to assess the expression stability of reference gene for various conditions prior to a complete study. Eukaryotic elongation factor 1 alpha (*eEF1A (s)*) and YT521**-**B**-**like family protein (*YT521-B*) have previously been identified as suitable reference genes for normalizing expression of target genes under different defoliation regimes in perennial ryegrass [31], but few similar studies assessing expression stability under other stresses have been published Therefore, for further development of qRT-PCR as a tool for studying transcriptional responses in perennial ryegrass, it is important to screen potential candidate reference genes under various abiotic stresses.

In this study, we contrasted the expression stability of 11 candidate reference genes (Zeitlupe [*ZTL*], 60S ribosomal protein [*60S*], Histone 3 [*H3*], *TEF1*, *GAPDH*, *UBQ*, *eEF1A (s)*, *TBP-1*, *eIF4A*, *YT521-B*, and *E2*) in perennial ryegrass under drought, heat, salt, waterlogging and ABA treatments. We used the results to identify the best reference genes for normalization of the expression of target genes in perennial ryegrass gene under each of these abiotic stresses.

## Materials and Methods

### Plant Materials, Growth Conditions and Stress Treatments

Perennial ryegrass cv. Barnauta were germinated and grown in 3**-**L pots containing 1 kg soil (soil pH of 5.18; organic qualitative content of 1.41%; N, P, K of 100.36 mg kg^−1^, 4.32 mg kg^−1^, and 337.24 mg kg^−1^, respectively) in a growth chamber at 25°C. A light source provided 100 μmol of photons m^−2^ s^−1^ on a 16/8**-**h light/dark regimen. Plants at the 6–8 leaf stage were used for all stress treatments. Control plants were watered every other day to maintain 80% soil water content, and each pot was watered and weighed every daily to maintain a total mass of 1 kg (0.97 kg potting medium plus 0.03 kg pot). The amount of water that added to each pot was recorded. Plants were exposed to a drought treatment by ending the above watering regime for 15 days. At the end of drought treatment, the leaf water content was 10% as measured by a method [32]. For heat treatment, plants were moved to a growth chamber set at 37°C for 7 days, and all other environmental conditions were held constant relative to the early growth stage. For salinity treatment, Plants were watered with 250 mmol L^−1^ NaCl for 12 days. For waterlogging treatment, Plants were flooded with water with 2.5 cm above the soil surface daily for 15 days, with control and treated pots maintained in the same growth chamber. For ABA treatment, Plants were treated with a concentration of 100 μmol L^−1^ ABA sprayed on the plants for 12 days, while control plants were treated with equal quantity of water. All experimental and control plants (leaves and roots) were separated three times with three biological replicates for expression analysis under different conditions.

### RNA Isolation and cDNA Synthesis

For all the experiments, RNA was extracted from plants using the Total RNA Kit II (Genebase Bioscience, China) according to the manufacturer’s instructions. Possible DNA contamination was removed from RNA samples by treatment with RNase**-**free DNase I provided in the kit. Concentration and purity of RNA samples were assessed spectrophotometrically with a NanoDrop ND**-**1000 Micro**-**Volume UV**-**Vis Spectrophotometer (NanoDrop Technologies, Wilmington, DE, USA), with 260/280 nm ratios in the range of 1.9 to 2.2 and 260/230 nm around 2.0 considered acceptable for use in qRT-PCR. Integrity of the RNA was also confirmed using 1% agarose gel electrophoresis. A total of 0.8 μg RNA was used for reverse transcription with an iScript cDNA Synthesis Kit (Bio**-**Rad Laboratories Inc., Hercules, California, USA) with Poly (A) and random primers in a 20****μl reaction volume according to the manufacturer’s instructions. The cDNA obtained for each sample was diluted 1∶20 with nuclease**-**free water for qRT-PCR.

### Primer Design and Quantitative RT-PCR Analysis

The *60S*, *GAPDH*, *UBQ*, *ZTL*, *TEF1*, peroxidase (*POD*), and superoxide dismutase (*SOD*) orthologous genes of wheat were used as ‘query’ to BLAST against available perennial ryegrass expressed sequence tags in the GenBank database (NCBI). All genes were named based on their similarity to known genes with sequence similarity from 90% to 98%. Primer sequences for an additional six reference genes’ were identified from a previous study [31].

Primers were designed using Primer 3 (http://frodo.wi.mit.edu/primer3/) using the following criteria: T_m_ between 58–62°C (optimum T_m_ of 60°C); PCR product size between 75–200 base pairs, length of 18 to 24 nucleotides in optimal length of 20 nucleotides) and GC content from 40% to 60%. In order to check the primers’ specificity, the PCR products were analyzed using 2.0% agarose gel electrophoresis and stained using Gelred (Biotium, USA).

QRT-PCR reactions were executed in 96**-**well blocks with a BIO**-**RAD CFX96 Real**-**Time PCR system (Bio**-**Rad, USA) using SYBR Premix Ex TaqTM (TaKaRa, Japan). The PCR reactions were 20 μl in volume and contained 10 μl 2×SYBR Premix Ex TaqTM, 2 μl diluted cDNA reaction mixture, 0.4 μl ROX Reference Dye II, and 1 μl 10 μM for each primer. The cycling conditions were as recommended by the manufacturer: 5 min at 95°C, 40 cycles of 95°C for 30 s, 58°C for 30 s, and 72°C for 30 s. At the end of the cycling process, the temperature was raised from 60°C to 95°C to obtain the dissociation curve. Three technical replicates were performed for each biological replicate – gene combination. The final cycle threshold (C_T_) values were calculated as the means of all nine values.

### Gene Expression Stability Analysis

The stability of reference gene expression was analyzed using four different VBA (Visual Basic Applet) applets: Delta C_T_ method [33], geNorm (ver. 3.5) [34], BestKeeper (ver. 1.0) [35] and Normfinder (ver. 0.953) [36]. Results from CFX manager (Bio Rad) were exported into Microsoft Excel 2007 and transformed to create input files for each target according to the requirements of each software. The geNorm tool ranked the reference genes by calculating the gene expression stability value (M_1_) based on the average pairwise expression ratio. The most stable reference gene has the lowest M_1_ value, while the least stable one presents the highest M_1_ value. The program considers M_1_ values below 1.5 to indicate stable expression. Normfinder is a Microsoft Excel application which ranks candidate genes according to stability index M_2_ based on the average pairwise variation of a given gene compared to all other studied genes [33]. The more stably expressed genes exhibit lower M_2_ values. BestKeeper is another Excel-based tool that identifies the most stably expressed genes by making comparisons of the coefficient of variance (CV) and the standard deviation (SD) [35]. The most stable genes are those with the lowest coefficient of variance and standard deviation (CV ± SD). Finally, the reference genes’ stability values derived from each of the three software tools were then used to create a comprehensive rank each of the 11 candidate reference genes in order from most to least stable expression under each abiotic stress condition using RefFinder [37]. This is a user**-**friendly, web**-**based tool developed for comprehensively evaluating and screening reference genes from extensive experimental datasets [37].

Gene**-**specific PCR efficiency was also calculated based on standard curves using a 10**-**fold serial dilution of the mixed cDNA template for each primer pair. The correlation coefficients (R^2^) and slope values were acquired from the standard curve. Calculate the coefficient of variation (CV) according to the equation: 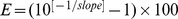 [38].

To detect the influence of reference genes on the outcome of an experiment, the relative expression patterns of two genes SOD and POD were evaluated using the most stable and unstable genes in Days 0, 3, 6 and 9 under drought treatment.

## Results

### Verification of PCR Amplicons, Primer Specificity, and PCR Amplification Efficiency

**Figure 1:**
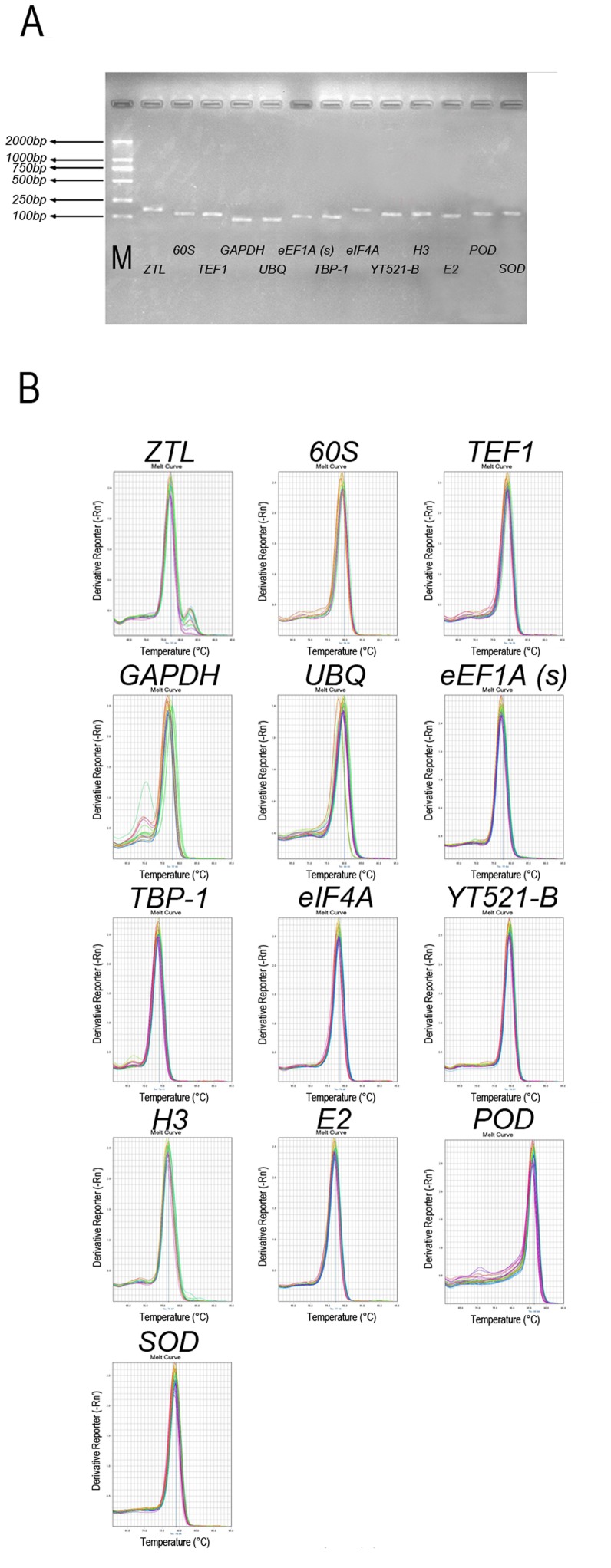
Primer specificity and amplicon size. (A) Agarose gel (2.0%) electrophoresis indicates amplification of a single PCR product of the expected size for 13 genes. (B) Melting curves of 13 genes show single peaks. M represents 100 bp DNA marker.

The description of 13 genes (11 candidate reference genes and 2 object genes), primer sets, melting temperatures (T_m_ values), and amplicon lengths assessed using qRT-PCR in this study are listed in Table 1. The melting temperatures (T_m_) values of all PCR products ranged from 74.11°C for *TBP* ***-*** *1* to 86.44°C for *POD* (Table 1). Amplion sizes were determined by testing each primer pair using electrophoresis on a 2% agarose gel (Figure 1A). This and the single peak melting curves of the qRT-PCR products (Figure 1B) demonstrated that a single PCR amplification product of the expected size was obtained for each reference gene. The amplification efficiency of all primers ranged from 103.02% (*eIF4A*) to 93.57% (*60S*; Table 1).

**Table 1:** List of primer sequences and related information for 13 genes (11 references gene and 2 object genes).

Genename	Gene function	ID	Primer sequence (5′→3′) (Forward)	Primer sequence (5′→3′) (Reverse)	T_m_(°C)	Amplicon Length (bp)	Amplificationefficiency (%)
ZTL	Zeitlupe	GR512712	AGTTCCAGGGTGATCTGCTG	TCACAACTTCCTTTGCCACA	77.19	180	98.64
60S	60S Ribosomal protein L18A	GR521461	GCAGAATACGAGGGCAATGT	ACCAAAGACACGGTTTCCAG	81.16	132	93.57
TEF1	Transcription elongation factor 1	GR522099	CGTGTGATCGAGAGGTTTGA	CGAATTTCCAGAGGGCAATA	79.78	133	96.09
GAPDH	Glyceraldehyde-3-phosphate dehydrogenase	GR518033	TCTGACCGTTAGACTTGAGAAGG	CTTGAGCTTACCCTCAGACTCCT	77.98	83	97.56
UBQ	Ubiquitin	GR510338	AAAATTCCCCAATCAATCTCCT	CTTCACAAAGATCTGCATCTTGA	80.08	89	96.04
eEF1A (s)	Eukaryotic elongation factor 1 alpha	GO924806	CCGTTTTGTCGAGTTTGGT	AGCAACTGTAACCGAACATAGC	77.64	113	101.02
TBP-1	26S proteasome regulatory subunit 6A homolog	GO924783	TGCTTAGTTCCCCTAAGATAGTGA	CTGAGACCAAACACGATTTCA	74.11	112	98.45
eIF4A	Eukaryotic initiation factor 4 alpha	GO924770	AACTCAACTTGAAGTGTTGGAGTG	AGATCTGGTCCTGGAAAGAATATG	78.49	168	103.02
YT521-B	YT521-B-like family protein	GO924780	TGTAGCTTGATCGCATACCC	ACTCCCTGGTAGCCACCTT	79.61	122	93.61
H3	Histone 3	GO924769	CACCAATGTTCTGCCTATCG	CAGACCAACGAACAAACGAC	76.67	135	99.09
E2	Ubiquitin-conjugating enzyme	GO924794	CGGTTCTGTGCCAAAATGT	CAGCTATCTCCAACGGTTCA	77.28	111	99.10
POD	Guaiacol peroxidase	GR521420	CTCTACAACGAGACCAACATCAA	GTAGACGTTGTCGAAGGAGTACG	86.44	135	95.34
SOD	Superoxide dismutase	GR521552	GTTGACAAGCATATCCCCCTTAC	AGTGCTCTTGCTAAGCTCATGTC	79.06	144	99.67

### Determination of C_T_ Values and Variation in Reference Gene Expression

**Figure 2:**
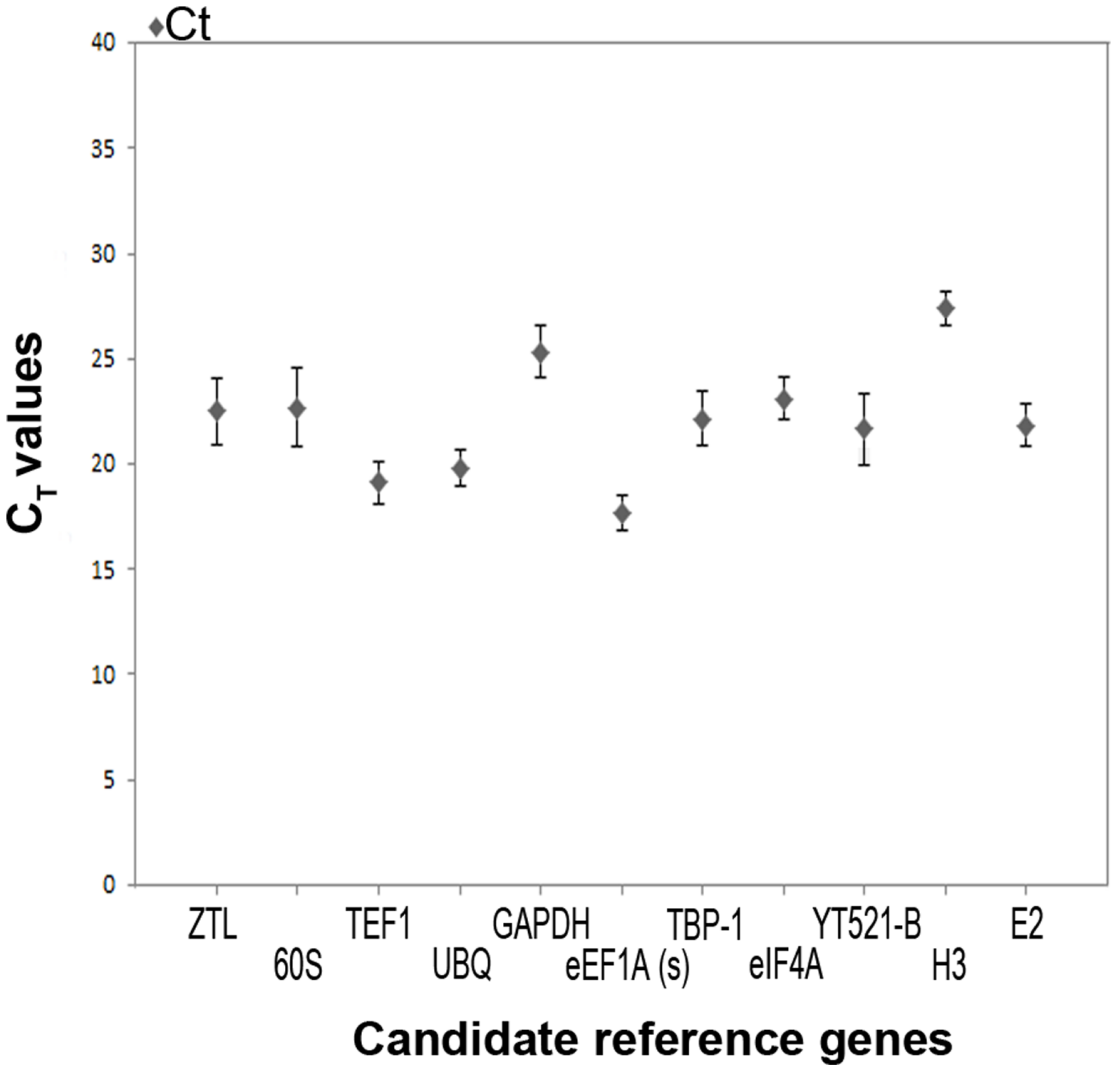
Median cycle threshold (C_T_) values for each reference gene for all samples. The filled diamond symbol indicates median C_T_ values. The bars indicate standard deviation.

Cycle threshold (C_T_) values were obtained from nine qRT-PCR reactions (three biological replicates, each with three technical replicates) for each of the 11 candidate reference genes under each of the abiotic stress treatments. To quantify overall differences in transcript levels for the 11 reference genes, we calculated the median C_T_ range, and CV of C_T_ for each gene across all samples. As expected, the median C_T_ values for each of the 11 candidate reference genes ranged from 15 to 30 cycles (Figure 2). Most displayed median C_T_ values ranging from 20 to 25, indicating a moderately high level of expression. The gene *eEF1A (s)* had the highest expression level among the tested candidate reference genes (i.e., had the lowest median C_T_ value), while *H3* had the lowest expression level (i.e. the highest median C_T_ values). To reveal the expression stability of candidate reference genes, it is necessary to assess the standard deviation of C_T_ values. In our results, some genes with high standard deviations (for instance, *ZTL*, *60S* and *YT521-B*) had relatively large standard deviations, indicating more variable expression levels, while others such as *UBQ*, *H3*, and *eIF4A* had smaller standard deviations and more stable expression patterns.

### Stability Ranking of Candidate Reference Genes

To compare stability of expression among the candidate reference genes, the computational methods, BestKeeper, Normfinder, and geNorm were applied to C_T_ values for each gene’s expression data. These tools are based on different models and assumptions and each produced different results for the same gene’s expression data. RefFinder was used to calculate a recommended comprehensive ranking based on the results of computational analysis, which in turn allowed us to identify the best reference genes for qRT-PCR data normalization in perennial ryegrass samples.

#### a) geNorm analysis

**Figure 3:**
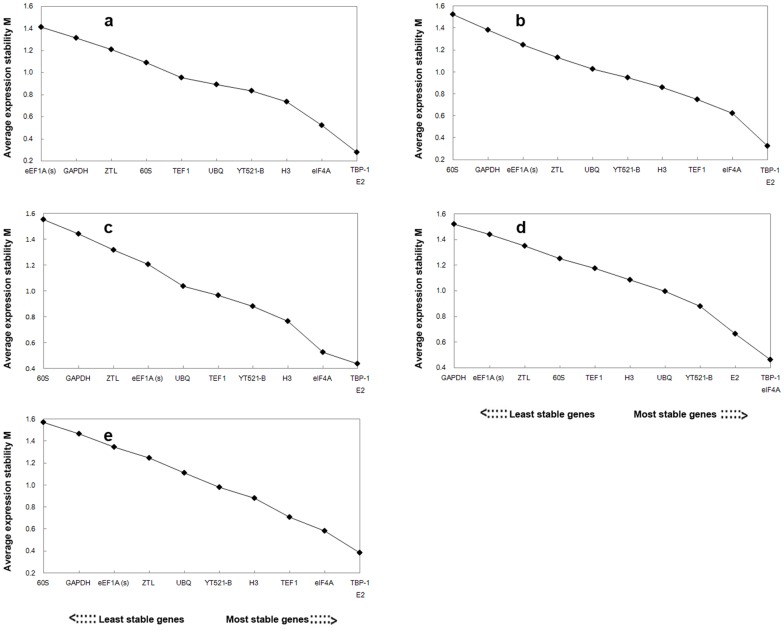
Average expression stability values (M_1_) of 11 candidate reference genes calculated by geNorm. (a) drought stress, (b) salt stress, (c) heat stress, (d) waterlogging stress, (e) ABA treatment. Lower M_1_ values indicate more stable expression.

We used geNorm to rank reference genes’ expression stability based on average pairwise expression ratios (M_1_), with values below 1.5 considered to represent stable expression. In our study, all genes except *60S* and *GAPDH* exhibited M_1_ values <1.5, indicating stable expression (Figure 3). Similar results were produced across different stress treatments. The *TBP-1*, *E2*, and *eIF4A* were top three reference genes under drought, salt, heat, waterlogging and ABA treatment.

To evaluate the optimal number of the reference genes required for accurate normalization, the pairwise variation between consecutively ranked genes (Vn/Vn+1) was calculated using geNorm. The optimal number of reference genes was identified as the lowest number of genes producing a pairwise variation of no more than 0.15 [34]. When all samples (i.e., across all stress treatments) were considered together to determine the optimal number of reference genes, the pairwise variation of V2/V3 was higher than 0.15 (0.173), the V3/V4 (0.145), indicating that three reference genes should be included for gene expression studies in perennial ryegrass that encompass multiple stress conditions.

#### b) Normfinder analysis

Normfinder ranks gene expression stability based on average pairwise variation of a gene compared to all other genes (M_2_). The expression stability calculated by Normfinder for each gene in this study is shown in Table 2. The results showed that *TEF1* and *UBQ* were most stable in expression, and therefore, they were the most appropriate reference genes, under drought and waterlogging stresses, with M_2_ values of 0.568 and 0.700, respectively. When considering salt, heat stresses, and ABA treatment, *eIF4A* was the most reliable reference gene, with M_2_ values of 0.628, 0.592, and 0.700, respectively. In contrast, *eEF1A (s)* was the least stable reference gene under drought and waterlogging stresses, and *60S* was the least stably expressed gene under salt, heat stresses, and ABA treatment.

**Table 2:** Expression stability values for perennial ryegrass candidate reference genes calculated using Normfinder under five treatments.

Rank	Drought stress	Salt stress	Heat stress	Waterlogging stress	ABA
1	TEF1 (0.568)	eIF4A (0.628)	eIF4A (0.592)	UBQ (0.700)	eIF4A (0.700)
2	eIF4A (0.573)	TEF1 (0.748)	YT521-B (0.757)	TEF1 (0.837)	TEF1 (0.718)
3	TBP-1 (0.698)	YT521-B (0.786)	TBP-1 (0.796)	YT521-B (0.847)	YT521-B (0.737)
4	UBQ (0.713)	TBP-1 (0.790)	H3 (0.839)	E2 (0.870)	H3 (0.754)
5	YT521-B (0.757)	H3 (0.799)	UBQ (0.920)	eIF4A (0.925)	E2 (0.990)
6	H3 (0.862)	UBQ (0.819)	TEF1 (0.931)	H3 (0.983)	UBQ (1.037)
7	E2 (0.924)	E2 (0.973)	E2 (0.939)	TBP-1 (1.031)	TBP-1 (1.085)
8	ZTL (1.224)	ZTL (1.080)	ZTL (1.340)	ZTL (1.209)	ZTL (1.240)
9	60S (1.483)	eEF1A (s) (1.592)	eEF1A (s) (1.559)	60S (1.403)	GAPDH (1.581)
10	GAPDH (1.516)	GAPDH (1.611)	GAPDH (1.563)	GAPDH (1.577)	eEF1A (s) (1.610)
11	eEF1A (s) (1.661)	60S (1.953)	60S (1.814)	eEF1A (s) (1.581)	60S (1.841)

Note: Expression stability and ranking of 11 candidate reference genes calculated with Normfinder under drought, salt, heat, waterlogging stresses and ABA treatment. Lower average expression stability (M_2_ value) indicates more stable expression.

#### c) BestKeeper analysis

BestKeeper analysis determined which genes exhibited the lowest (CV±standard deviations) in order to judge stability of gene expression (Table 3). Results indicate that *ZTL* had values of 1.02±0.02, 1.32±0.03, 0.85±0.05, 1.23±0.03, and 1.32±0.09 under drought, salt, heat, waterlogging, and ABA treatments, respectively, making it the most stably**-**expressed reference gene in all cases. However, *60S* had CV values of 6.14±1.83, 7.29±2.21, 6.35±1.89, 6.04±1.78 and 6.68±1.99, respectively, under the same abiotic stresses, making it the least stable. The results obtained from BestKeeper indicated that few differences occurred across stress treatments (Table 3).

**Table 3:** Expression stability values for perennial ryegrass candidate reference genes calculated using BestKeeper under five treatments.

Rank	Drought	Salt stress	Heat stress	Waterlogging stress	ABA
1	ZTL (1.02±0.02)	ZTL (1.32±0.03)	ZTL (0.85±0.05)	ZTL (1.23±0.03)	ZTL (1.32±0.09)
2	TEF1 (2.35±0.70 )	TEF1 (2.04±0.61)	eEF1A (s) (2.94±0.92)	TEF1(1.71±0.51)	TEF1 (3.17±0.94)
3	eEF1A (s) (2.55±0.81)	eEF1A (s) (2.17±0.69)	GAPDH (3.79±1.04)	eEF1A (s) (2.19±0.69)	eEF1A (s) (3.11±0.97)
4	GAPDH (3.75±1.03)	eIF4A (2.83±0.85)	H3 (4.27±1.22)	GAPDH (3.99±1.09)	GAPDH (3.89±1.06)
5	eIF4A (3.72±1.10)	TBP-1 (3.49±0.96)	TEF1 (5.74±1.53)	E2 (3.96±1.30)	H3 (4.42±1.26)
6	UBQ (4.84±1.36)	E2 (3.46±1.15)	eIF4A (4.53±1.33)	H3 (4.76±1.36)	eIF4A (4.42±1.30)
7	TBP-1 (5.13±1.38)	H3 (4.40±1.28)	E2 (4.48±1.47)	eIF4A (4.67±1.37)	E2 (4.25±1.40)
8	H3 (4.83±1.38)	YT521-B (4.35±1.34)	TBP-1 (5.74±1.53)	UBQ (4.94±1.38)	TBP-1 (5.24±1.41)
9	YT521-B (5.05±1.55)	GAPDH (4.85±1.35)	UBQ (6.03±1.66)	TBP-1 (6.17±1.64)	UBQ (5.47±1.52)
10	E2 (4.88±1.60)	UBQ (5.01±1.42)	YT521-B (5.47±1.67)	YT521-B (5.39±1.64)	YT521-B (5.10±1.56)
11	60S (6.14±1.83)	60S (7.29±2.21)	60S (6.35±1.89)	60S (6.04±1.78)	60S (6.68±1.99)

Note: Expression stability and ranking of 11 candidate reference genes calculated with BestKeeper under drought, salt, heat, waterlogging stresses and ABA treatment. Eleven reference genes are identified as the most stable genes, as evaluated by the lowest values of the coefficient of variance (CV) and standard deviation (SD).

#### d) RefFinder analysis

Finally, we used RefFinder to create a comprehensive ranking of the most stably expressed candidate reference genes within each experimental treatment condition (Table 4).

**Table 4:** Stability ranking of 11 candidate reference genes.

Methods	A. Ranking Order under drought stress (Better–Good–Average)										
	1	2	3	4	5	6	7	8	9	10	11
Delta C_T_	eIF4A	TBP-1	TEF1	UBQ	YT521-B	H3	E2	ZTL	60S	GAPDH	eEF1A (s)
BestKeeper	ZTL	TEF1	eEF1A (s)	GAPDH	eIF4A	UBQ	TBP-1	H3	YT521-B	E2	60S
Normfinder	TEF1	eIF4A	TBP-1	UBQ	YT521-B	H3	E2	ZTL	60S	GAPDH	eEF1A (s)
geNorm	TBP-1 | E2		eIF4A	H3	YT521-B	UBQ	TEF1	60S	ZTL	GAPDH	eEF1A (s)
Recommended comprehensive ranking	**eIF4A**	**TEF1**	**TBP-1**	**E2**	**UBQ**	**ZTL**	**YT521-B**	**H3**	**eEF1A (s)**	**GAPDH**	**60S**
	**B. Ranking Order under salt stress (Better–Good–Average)**										
Delta C_T_	eIF4A	TBP-1	TEF1	YT521-B	H3	UBQ	E2	ZTL	eEF1A (s)	GAPDH	60S
BestKeeper	ZTL	TEF1	eEF1A (s)	eIF4A	TBP-1	E2	H3	YT521-B	GAPDH	UBQ	60S
Normfinder	eIF4A	TEF1	YT521-B	TBP-1	H3	UBQ	E2	ZTL	eEF1A (s)	GAPDH	60S
geNorm	TBP-1 | E2		eIF4A	TEF1	H3	YT521-B	UBQ	ZTL	eEF1A (s)	GAPDH	60S
Recommended comprehensive ranking	**eIF4A**	**TBP-1**	**TEF1**	**E2**	**ZTL**	**YT521-B**	**H3**	**eEF1A (s)**	**UBQ**	**GAPDH**	**60S**
	**C. Ranking Order under heat stress (Better–Good–Average)**										
Delta C_T_	eIF4A	TBP-1	YT521-B	E2	H3	TEF1	UBQ	ZTL	eEF1A (s)	GAPDH	60S
BestKeeper	ZTL	eEF1A (s)	GAPDH	H3	TEF1	eIF4A	E2	TBP-1	UBQ	YT521-B	60S
Normfinder	eIF4A	YT521-B	TBP-1	H3	UBQ	TEF1	E2	ZTL	eEF1A (s)	GAPDH	60S
geNorm	TBP-1 | E2		eIF4A	H3	YT521-B	TEF1	UBQ	eEF1A (s)	ZTL	GAPDH	60S
Recommended comprehensive ranking	**eIF4A**	**TBP-1**	**E2**	**YT521-B**	**H3**	**ZTL**	**TEF1**	**eEF1A (s)**	**UBQ**	**GAPDH**	**60S**
	**D. Ranking Order under waterlogging stress (Better–Good–Average)**										
Delta C_T_	UBQ	YT521-B	E2	eIF4A	TEF1	TBP-1	H3	ZTL	60S	eEF1A (s)	GAPDH
BestKeeper	ZTL	TEF1	eEF1A (s)	GAPDH	E2	H3	eIF4A	UBQ	TBP-1	YT521-B	60S
Normfinder	UBQ	TEF1	YT521-B	E2	eIF4A	H3	TBP-1	ZTL	60S	GAPDH	eEF1A (s)
geNorm	TBP-1 | eIF4A		E2	YT521-B	UBQ	H3	TEF1	60S	ZTL	eEF1A (s)	GAPDH
Recommended comprehensive ranking	**UBQ**	**TEF1**	**eIF4A**	**E2**	**YT521-B**	**TBP-1**	**ZTL**	**H3**	**eEF1A (s)**	**GAPDH**	**60S**
	**E. Ranking Order under ABA treatment (Better–Good–Average)**										
Delta C_T_	eIF4A	TEF1	YT521-B	H3	E2	TBP-1	UBQ	ZTL	eEF1A (s)	GAPDH	60S
BestKeeper	ZTL	TEF1	eEF1A (s)	GAPDH	H3	eIF4A	E2	TBP-1	UBQ	YT521-B	60S
Normfinder	eIF4A	TEF1	YT521-B	H3	E2	UBQ	TBP-1	ZTL	GAPDH	eEF1A (s)	60S
geNorm	TBP-1 | E2		eIF4A	TEF1	H3	YT521-B	UBQ	ZTL	eEF1A (s)	GAPDH	60S
Recommended comprehensive ranking	**eIF4A**	**TEF1**	**E2**	**TBP-1**	**H3**	**ZTL**	**YT521-B**	**eEF1A (s)**	**UBQ**	**GAPDH**	**60S**

Under drought stress, RefFinder’s component programs identified five genes (*eIF4A*, *TEF1*, *TBP-1*, *E2*, and *UBQ*) as potential suitable reference genes. The three best reference genes were *eIF4A*, *TEF1*, and *TBP-1* (Table 4A). With respect to salt stress, a similar group of five genes (*eIF4A*, *TBP-1*, *TEF1*, *E2*, and *ZTL*) were identified as potential reference genes, and the same three genes identified as most stable under drought stress were again the three most stable reference genes in roots and leaves of salt**-**stressed perennial ryegrass (Table 4B). The five most suitable reference genes identified for use in heat stress experiments were *eIF4A*, *TBP-1*, *E2*, *YT521-B*, and *H3*, with the first three of these (*eIF4A*, *TBP-1*, and *E2*) the most stably expressed (Table 4C). Under waterlogging treatment, five genes (*UBQ*, *TEF1*, *eIF4A*, *E2*, and *YT521-B*) were identified as potential suitable reference genes based on the component programs of RefFinder. *UBQ*, *TEF1*, and *eIF4A* were the three most stable reference genes in both roots and leaves (Table 4D). Under ABA treatment, the component programs identified different gene pairs as the most stably expressed: *eIF4A* and *TEF1* for Delta C_T_ and Normfinder, *ZTL* and *TEF1* for BestKeeper, and *TBP-1* and *E2* for geNorm. RefFinder’s comprehensive ranking identified *eIF4A*, *TEF1*, and *E2* as the overall best three reference genes (Table 4E). Finally, under different abiotic stress treatments overall, *60S* and *GAPDH* were found to be last two least stably expressed reference genes using most of programs (Table 4).

### Validation of the Usefulness of the Reference Genes Identified from this Study

**Figure 4:**
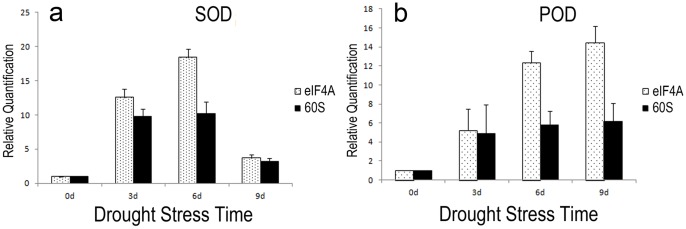
Expression levels of *SOD* (a) and *POD* (b) in different time (Days 0, 3, 6, and 9) of perennial ryegrass leaves under drought stress. Genes were normalized to highly stable (*eIF4A*) and unstable (*60S*) reference genes. Error bars indicate one standard error of the mean.

**Figure 5:**
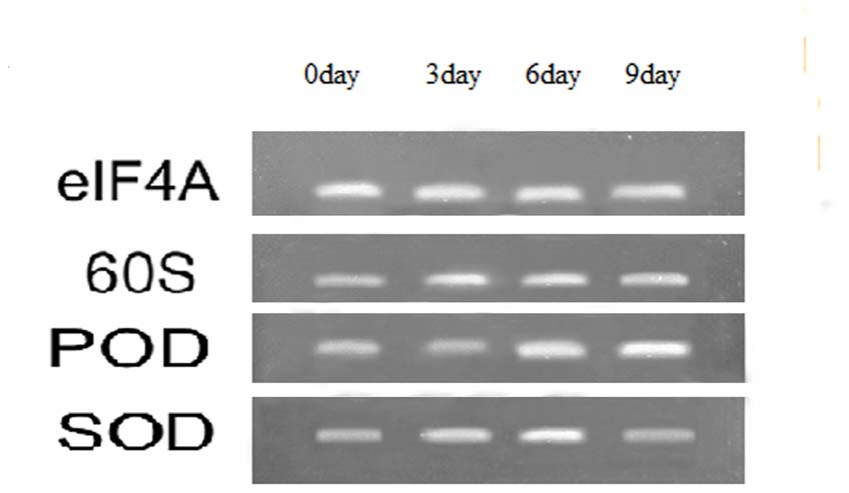
Agarose gel results of *SOD*, *POD*, *elF4A*, and *60S* PCR amplicons in perennial ryegrass leaves exposed to drought stress treatment.

To validate the performance of the reference genes identified in this study on known abiotic-stress inducible genes, we quantified the expression of *SOD* and *POD* genes which are up-regulated under dehydration drought stress [39]–[41]. After quantifying, a representative least stable reference gene (*60S*) and a representative most stable reference gene (*eIF4A*) were used to normalize their expression. As shown in Figure 4, using *60S* for normalization, suggests that both *SOD* and *POD* genes are induced ten or six fold, respectively, on both Day 3 and Day 6 after initiation of drought treatment. In contrast, using *eIF4A* as the reference gene reveals greater overall fold changes in expression of *SOD* and *POD* compared to Day 0 and greater expression of both genes on Day 6 than on Day 3. Furthermore, if *60S* was used as reference gene, we would have consistently failed to detect drought**-**induced gene expression changes in perennial ryegrass leaves for *SOD* (Day 3 to Day 6) and *POD* (between Days 3, 6, and 9) that were clearly identified when normalization was carried out with respect to *eIF4A* (Figure 4). Gel electrophoresis of PCR products also showed (Figure 5) that *eIF4A* was expressed more stable than *60S* under drought treatment.

## Discussion

Because of its high sensitivity, specificity and broad quantification range, qRT-PCR has significantly improved the quantification and detection of gene expression differences in distinct biological samples [42]–[44]. Quantification of gene expression is affected by several factors, such as RNA quality, efficiency of reverse transcription, cDNA quantity, expression of reference genes, and statistical methods [35], [45]. The selection of reference genes is an important step; because they are the basis for comparison of expression changes in target genes and it is necessary to select suitable, stably**-**expressed reference genes for each experiment [46]. In this study, expression stability was evaluated for 11 perennial ryegrass genes under five stress treatments to determine which are the most suitable for experimental use. While no single gene had perfectly stable expression under all treatment conditions, the candidates showed differing levels of stability allowing preference rankings for different types of experiments. Validating the expression stability of candidate reference genes in the species and treatments of interest before qRT-PCR normalization is preferable to use published reference genes without such initial screening.

Given the differences among rankings from the four computational methods used to determine expression stability, some overall patterns based on different stress treatments could be identified. In particular, *eIF4A* was the most stable reference gene under drought, salt, heat stresses, and ABA treatment, but not under waterlogging stress (Table 4). This gene has previously been identified as a stable reference gene in *Arabidopsis*, rice (*Oryza sativa*) and *Lolium perenne* [47]–[49]. However, *eIF4A* has performed poorly as a reference gene in oats (*Avena sativa*) and barley (*Hordeum vulgare*), so its use as a reference gene which is not recommended for these species [50]. Therefore, we believe that good reference genes should be selected based on different species or different stress treatment involved. Similarly, *UBQ* witnessed the most stable expression under waterlogging treatment in this study (Table 4E) and has previously performed well in the developing seeds of Tung tree (*Vernicia fordii* Hemsl.) [22] and *Arabidopsis* [51]. Nevertheless, recent studies have shown that *UBQ* reference gene can have unstable expression under other conditions [30], [47] and should not be used as an internal control gene in rice or soybean (*Glycine max*) [48], [52]. Although traditional housekeeping function genes are often considered good candidate reference genes due to their functional nature [53]–[55], some studies have found that these genes are frequently unstable. For example, Hong et al. [56] selected *GAPDH* as a stably expressed gene under various abiotic stress conditions in *Brachypodium distachyon*, but in our analyses, the stability of *GAPDH* ranked much lower than other reference genes under a variety of abiotic stresses (Table 4A). Previous studies have also demonstrated that *GAPDH* has unstable expression in rice and *Nicotiana tabacum* [48], [57], which may be due to species**-**dependent differences in gene expression.

In our study, we applied four commonly used programs (geNorm, Normfinder, BestKeeper, and Delta C_T_) to analyze the stabilities of all the 11 candidate reference genes. However, we got different results using different programmers. After analyzing the qRT-PCR data under drought treatment, Delta C_T_ identified that *eIF4A* was the most stable reference gene, followed by *TBP-1* and *TEF1*. BestKeeper identified that *ZTL* was the most stable reference genes, followed by *TEF1* and *eEF1A (s)*. Normfinder identified *TEF1* was the most stable reference gene, followed by *eIF4A* and *TBP-1*. GeNorm identified *TBP-1* and *E2* were the two most stable reference genes, followed by *E2*. *60S* was the least stable reference gene assessed by all four programs, so it is not recommended for using in perennial ryegrass abiotic stress genes expression studies. There were some different rankings of the reference gene’s stabilities using these four methods. This inconsistency may imply differences among the statistical algorithms. However, in this study, the unsuitable genes (*60S; GAPDH*) were ranked more consistently by using these four methods may due to the wide expression variability of these unsuitable genes. Some other studies also confirmed the above**-**mentioned results [22], [58]. Thus, a comparison of different algorithms of reference gene selection may allow a more precise evaluation of the most stable reference genes and reduce the risk of selection of co**-**regulated transcripts [59].

The selection of reference genes is an important step in establishing appropriate controls for qRT-PCR, and it is necessary to select suitable reference genes for each experiment. In the current study, *eIF4A*, *TEF1*, and *TBP-1* were the three best reference genes for drought stress experiments in perennial ryegrass; these three genes also performed well under salt stress, although the expression stability rankings of the latter two genes were switched. For heat stress experiments, *eIF4A*, *TBP-1*, and *E2* were the three most stably expressed reference genes, while *UBQ*, *TEF1*, and *eIF4A* were best reference genes to study waterlogging stress. Finally, under ABA treatment, *eIF4A*, *TEF1*, and *E2* showed the most stable expression. These genes are therefore recommended as suitable reference genes for each type of abiotic stress study in perennial ryegrass. The methods and results of this study can aid the accurate quantification of target genes of perennial ryegrass and other plants.
